# EMG-based vibro-tactile biofeedback training: effective learning accelerator for children and adolescents with dystonia? A pilot crossover trial

**DOI:** 10.1186/s12984-019-0620-y

**Published:** 2019-11-27

**Authors:** Claudia Casellato, Emilia Ambrosini, Andrea Galbiati, Emilia Biffi, Ambra Cesareo, Elena Beretta, Francesca Lunardini, Giovanna Zorzi, Terence D. Sanger, Alessandra Pedrocchi

**Affiliations:** 1 NearLab, Department of Electronics, Information and Bioengineering, Politecnico di Milano, Milan, Italy; 2 Department of Brain and Behavioral Sciences, University of Pavia, Pavia, Italy; 3 Scientific Institute, IRCCS E. Medea, Lecco, Bosisio Parini Italy; 4 Department of Child Neurology, Foundation IRCCS Neurological Institute Carlo Besta, Milan, Italy; 5 Department of Biomedical Engineering, University of Southern California, Los Angeles, USA; 6 Department of Neurology, Children Hospital of Los Angeles, Los Angeles, USA

**Keywords:** Dystonia, Biofeedback, EMG, Learning, Wearable devices, Sensory-motor deficits

## Abstract

**Background:**

This study is aimed at better understanding the role of a wearable and silent ElectroMyoGraphy-based biofeedback on motor learning in children and adolescents with primary and secondary dystonia.

**Methods:**

A crossover study with a wash-out period of at least 1 week was designed; the device provides the patient with a vibration proportional to the activation of an impaired target muscle. The protocol consisted of two 5-day blocks during which subjects were trained and tested on a figure-8 writing task: their performances (at different levels of difficulty) were evaluated in terms of both kinematics and muscular activations on day 1 and day 5, while the other 3 days were purely used as training sessions. The training was performed with and without using the biofeedback device: the week of use was randomized. Data were collected on 14 subjects with primary and secondary (acquired) dystonia (age: 6–19 years).

**Results:**

Results comparing kinematic-based and EMG-based outcome measures pre- and post-training showed learning due to practice for both subjects with primary and secondary dystonia. On top of said learning, an improvement in terms of inter-joint coordination and muscular pattern functionality was recorded only for secondary dystonia subjects, when trained with the aid of the EMG-based biofeedback device.

**Conclusions:**

Our results support the hypothesis that children and adolescents with primary dystonia in which there is intact sensory processing do not benefit from feedback augmentation, whereas children with secondary dystonia, in which sensory deficits are often present, exhibit a higher learning capacity when augmented movement-related sensory information is provided. This study represents a fundamental investigation to address the scarcity of noninvasive therapeutic interventions for young subjects with dystonia.

## Background

Dystonia is defined as a movement disorder in which involuntary sustained or intermittent muscle contractions cause twisting and repetitive movements, abnormal postures, overflow and co-contractions [1, 2]. In terms of etiology, dystonia is classified as primary when it is the most important feature of an idiopathic or an identified genetic disorder [3], while secondary dystonia are symptomatic disorders arising from another underlying disease, such as cerebral palsy (CP) or acquired brain injury. The term “secondary dystonia” as used in this work corresponds most closely to the concept of “acquired” dystonia defined in the more recent classification [2].

Among the available interventions to treat the motor symptoms, there are pharmacological, physical and occupational therapies, which are only partially successful, or deep brain stimulation, which is invasive and not necessarily effective, particularly for secondary dystonia [4–6]. Therefore, new noninvasive options for treating dystonia are strongly needed [7, 8]. Promoting strategies to learn a better execution of motor tasks has the potential to reduce the impact of motor symptoms in the daily life of these children [9, 10]. The learning process is strongly affected by sensory feedback, suggesting that interventions affecting sensory function may be beneficial for motor disorders. The theory of failure of motor learning [11] provides a mathematical model in which sensory deficits can prevent motor learning. An important prediction of the theory is that further improvement is possible through practice only if sensory deficits are corrected. We hypothesize that when sensory deficits are present during the period of motor development in childhood, there may be ongoing reduced motor function due to interference with learning, yet there remains the opportunity for subsequent improvement in motor learning and motor function if the sensory deficit can be reversed.

The pathophysiology of dystonia is varied; there is evidence that subjects with primary dystonia do not show sensory deficits, whereas subjects with secondary dystonia are frequently characterized by sensory abnormalities [12, 13]. Therefore the theory of motor learning hypothesizes that children with secondary dystonia who have sensory deficits may have a potentially reversible component of their motor deficit due to sensory interference with motor learning [11, 13–15]. This theory makes the prediction that reversal of sensory deficits at any age may remove the barrier to learning and improve motor function. One possible mechanism for improvement of sensorimotor functionality is represented by biofeedback techniques, which provide the subject with augmented task-relevant sensory information. Vibro-tactile feedback, alone or in combination with auditory signal, showed to improve motor performance and spatial perception in healthy [16, 17]. Furthermore, auditory feedback of body movements has recently shown to prevent spatial development delays in visually impaired children [18].

Most of the studies investigating the effects of biofeedback therapy in children and adolescents with CP and secondary dystonia reported a general positive effect [8, 19–24], with improvements in motor control, mobility, and motivation to practice; however, some limitations still need to be overcome. First, some of the studies [21, 22] employed a visual biofeedback which acts as an extrinsic feedback via external pathways, in contrast to intrinsic feedback which develops through proprioceptive pathways during movement. Secondly, a continuous visual feedback with a wearable device is more intrusive for use outside clinical or laboratory settings, such as school or domestic environments characterized by social interactions. Thirdly, the effectiveness of the biofeedback techniques was assessed on few subjects (2 or 3 participants in [19, 20, 24]) or using only qualitative interviews and clinical scales, without any quantitative measures able to capture small motor changes [23]. Lastly, comparisons about the effect of biofeedback training on subjects with primary and secondary dystonia were not reported so far.

Based on these premises, our prediction is that children with secondary dystonia would benefit from sensory augmentation provided by the biofeedback. On the other hand, we predict that children with primary dystonia will not show any specific improvement from the use of our system in terms of learning, since they are generally free from sensory deficits.

To verify this hypothesis, we designed a crossover multi-center study in order to quantitatively test the efficacy of an electromyographic (EMG)-based vibro-tactile biofeedback device for accelerating motor learning and improving motor skills in children and adolescents with both primary and secondary dystonia. The biofeedback signal was generated by a battery-powered wearable device, suitable for use during daily life activities, where the rotation speed of a silent vibration motor is set proportional to the level of muscle electrical activity; this device was preliminarily tested on children with secondary dystonia, showing promising effects on motor learning [23, 24]. The present work reports the results comparing 14 children and adolescents with primary and secondary dystonia. The performance of healthy age-matched subjects was evaluated to quantify the degree of normalization of function that can be achieved. To address the need for sensitive outcome measures, we exploited quantitative outcome measures designed and validated in previous studies in order to objectively assess performance and learning: these measures couple kinematic parameters, which describe the whole upper limb motion, and EMG activations related to the generated kinematics [25, 26].

## Methods

### Study design

This is a multi-center crossover study composed by 2 weeks of training with a wash-out period of minimum 1 to maximum 4 weeks. The weekly training was performed with or without the use of the biofeedback device. Primary dystonia subjects were recruited at the Neurological Institute IRCCS C. Besta, Milano, Italy and performed the training at Politecnico di Milano. Secondary dystonia subjects were instead recruited and trained at the Scientific Institute E. Medea. Healthy subjects were recruited and tested at Politecnico di Milano. The protocol of the study was approved by the Ethical Committees of the Scientific Institute E. Medea (reference number: 054/14-CE; Date: 01-04-2015) and of the Neurological Institute IRCCS C. Besta, Milano, Italy (reference number: 24; Date: 16-12-2015), and was performed in accordance with the Declaration of Helsinki.

### Study protocol

**Fig. 1:**
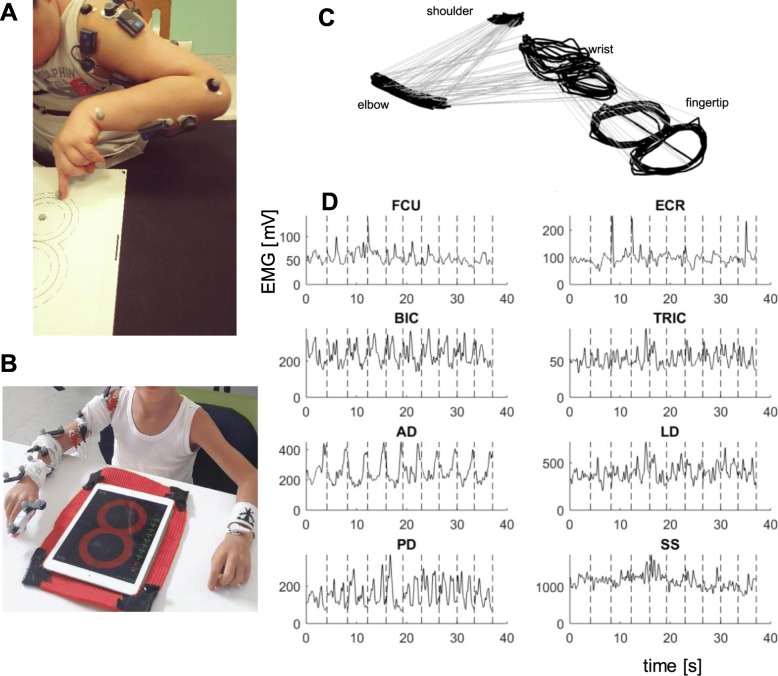
Experimental set-up and raw data. **a**) a secondary dystonia subject performing the experiment at Scientific Institute E. Medea. **b**) a primary dystonia subject performing the experiment at Politecnico di Milano. **c** and **d** an example of recorded dataset: 10-movement sequence of the figure-8 task, performed by a healthy subject at 30 bpm: 3D kinematics (**c**) and EMG envelopes (**d**). Vertical dashed lines identify every figure-8 repetitions. Flexor Carpi Radialis (FCR), Extensor Carpi Radialis (ECR), Biceps Brachii (BIC), Triceps Brachii (TRIC), Anterior Deltoid (AD), Lateral Deltoid (LD), Posterior Deltoid (PD), and Supraspinatus (SS)

The training consisted in performing a figure-8 writing task, relevant to daily life, using the dominant side. Subjects were provided with a guideline figure-8 trace on a tablet computer (primary dystonia and healthy) or on paper (secondary dystonia). The figure-8 trace on the tablet (iPad, Apple) was composed of two circles with a radius of 4 cm each (Fig. 1b). When paper was used, the same shape and size of the figure-8 trace was drawn, if feasible (Fig. 1a); for the most impaired subjects, a larger size was used. All participants were instructed to use their index fingertip to follow the trace with the maximum accuracy while maintaining a pre-defined speed.

The experiment consisted of two 5-day blocks, performed in randomized order (using a list of codes previously generated through a permuted-block randomization procedure; an automatic assignment system, developed in MATLAB, was used to conceal the allocation). Each 5-day block was composed of two testing days (Day 1 and Day 5) and three training days (Days 2, 3 and 4). The first day of the first block, three difficulty levels (speed values) were identified for each subject. The identification was carried out by preliminary tests, during which the subject was asked to match a target speed for at least 5 repetitions in a row. The objective was to set the levels as challenging but achievable. During the testing days, the subject performed a sequence of 17 continuous figure-8 movements for each target speed; the first 7 repetitions were performed with a metronome to impose the pace, then the metronome was switched off and the subject was asked to autonomously maintain the same pace. The 10-movement sequence without acoustic cue was then considered for data analysis (regardless if the intended speed was actually maintained). During the testing days, the biofeedback device was never used. During the training days, subjects were asked to practice by repeating multiple sequences of figure-8 movements, at the intermediate target speed, for about 30 min. During the training days of one block, the task was performed with the biofeedback device (BF+), while during the other block it was performed without the device (BF-). During the BF+ block, we did not ask the subjects to match a specific level of muscle contraction, letting the biofeedback steer the awareness.

### Participants

Inclusion criteria were: i) primary or secondary dystonia affecting the dominant arm; ii) developmental age (6–20 years); iii) no cognitive impairment that prevents understanding of instructions; iv) a stable drug therapy during the investigation; v) no treatment with botulinum toxin in the dominant arm in the 6 months prior to recruitment.

At the beginning of the first block, participants were involved in a baseline assessment to quantify dystonia severity in the dominant upper limb based on the Barry-Albright Dystonia Scale (BAD), which ranges for 0 (absent) to 4 (severe).

In order to obtain healthy reference values for all the outcome measures, a group of age-matched healthy subjects were recruited and involved in the protocol of a single testing day, therefore without the use of BF.

All participants gave informed written consent for participation. In case of minors, parents were asked to sign the informed consent and the authorization for use of protected health information, videos and images.

### Experimental apparatus

A 3-dimensional motion-tracking system was used to record the subject’s movement. Passive markers were placed on the shoulder, elbow, wrist joints, and on the index fingertip (Fig. 1). Different commercial systems were used at each of the two sites. At Politecnico di Milano, where primary dystonia and healthy subjects were collected: POLARIS VICRA (sampling frequency of 20 Hz); at Medea Institute, where secondary dystonia subjects were recruited: OEP System, BTS Bioengineering (sampling frequency of 60 Hz). When the tablet was used (at Politecnico di Milano), the 2D coordinates of the index fingertip were also recorded by an ad-hoc touch-based application (2D touch coordinates at a sampling frequency of 60 Hz).

The muscular activity was recorded using a multi-channel EMG amplifier. Bipolar surface EMG electrodes were positioned on eight muscles of the upper limb: Flexor Carpi Radialis (FCR), Extensor Carpi Radialis (ECR), Biceps Brachii (BIC), Triceps Brachii (TRIC), Anterior Deltoid (AD), Lateral Deltoid (LD), Posterior Deltoid (PD), and Supraspinatus (SS). Different commercial EMG systems were used at each site. At Politecnico di Milano (primary and healthy): Porti 32 TMSi (sampling frequency of 2048 Hz); at Medea Institute (secondary): BTS Free EMG (sampling frequency of 1000 Hz).

**Fig. 2:**
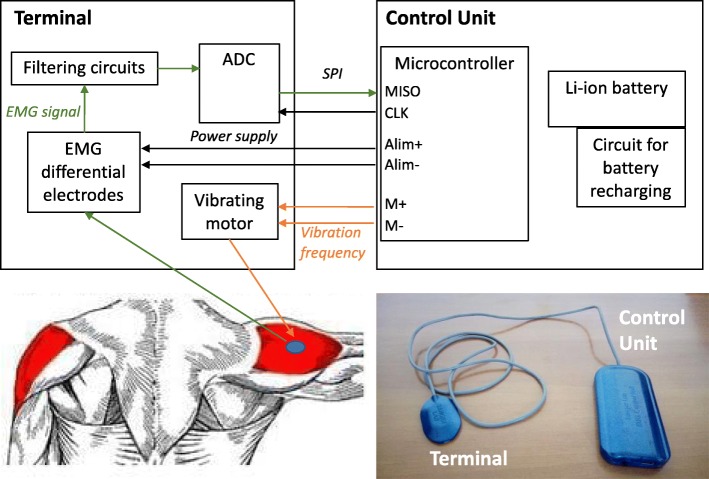
Biofeedback device. Picture and block scheme of the biofeedback device

During the training days of the BF+ block, the subject was asked to wear the EMG-based vibro-tactile biofeedback device on a target muscle of the dominant arm. For each patient, based on a clinical examination, the target muscle was selected among the 8 recorded muscles as the one whose activity mostly interefered with the upper limb functionalities (e.g. self-feeding, writing). Clinicians from both sites reviewed videotapes in order to verify appropriate choice at study entry. The device consists of an electrode head (*terminal*, Fig. 2) connected to a belt pack (*Control unit*, Fig. 2). The terminal contains an active differential surface electrode to record the EMG activity of the target muscle, and a vibration motor, so that the feedback occurs directly at the site of the target muscle, making the stimulus salient and relevant. The Control unit computes the amplitude of the EMG signal of the target muscle through Bayesian estimation [27] and actuates a silent vibration motor with a rotation speed and amplitude proportional to the magnitude of the EMG. The fast processor and the use of a nonlinear filter allow the device to implement online proportional biofeedback.

### Data analysis

Data collected during the testing days (Day 1 and Day 5) of each block (BF+ and BF-) were analyzed. Data analysis was executed in Matlab R2016a (The Mathworks, Natick, MA, USA).

Kinematic data of each joint were projected on the movement plane by Principal Component Analysis (PCA), after verifying that the plane formed by the first 2 Principal Components (PC) always contained more than 95% of joint 3D data variance. Within each 10-movement sequence (each Day, each Block, and each target speed), single figure-8 repetitions were identified.

EMG data were high-pass filtered (Butterworth, 5th order, cutoff frequency of 10 Hz), rectified, and finally low-pass filtered (Butterworth, 5th order, cutoff frequency of 5 Hz) to extract envelopes.

From the pre-processed kinematic, the following outcome measures were derived for each single figure-8 repetition:

i. Time*Error. It represents a synthetic index of the speed-accuracy trade-off (*SATO*). It was computed as the product between the accuracy error (*Norm Error*) and the movement time (*Norm Time*), where the accuracy error was calculated as the average, over time frames, of the absolute distance between the fingertip and the desired path, normalized to the trace width; and the movement time was computed as the actual duration of each repetition, normalized to the maximum duration obtained by each subject across all repetitions of all sessions [25, 28]. With learning, this index should be tuned, e.g. by a down-shift of the trade-off (decreased error with equal movement time or decrease movement time with equal error).
ii. Coefficient of variation of the 2D finger speed (*CV*_*speed*_). It was computed as the ratio between the standard deviation and the mean value of the 2D finger speed [29]. With learning, this index should decrease as an effect of the reduction of the speed changes, thus corresponding to an increased smoothness.
iii. Kinematic dissimilarity. Procrustes analysis was applied to find out the optimal linear transformation (translation, reflection, orthogonal rotation, and scaling) able to map wrist, elbow and shoulder joints on the end effector (finger) in terms of 2D coordinates (*Diss*_*WR*_, *Diss*_*EL*_ and *Diss*_*SHO*_ for wrist, elbow and shoulder, respectively). From distal to proximal, a progressive physiological loss of «output shape» should occur, which corresponds to an increase of kinematic dissimilarity [30]. With learning, these values should decrease, towards a more functional and synergic motion along the whole arm chain.

From the pre-processed kinematic and EMG data, the following outcome measures were instead derived from the whole 10-movement sequence of each single repetition:

i. *Repeatability*. It was computed as the variance (%) explained by the first PC applied on the 2D finger trajectories of each repetition, after time-normalization on the mean duration across all repetitions. With learning, this index should increase.
ii. *Task-Correlation-Index* (*TCI*). For each EMG channel, the EMG spectrum was computed by Fourier analysis on the EMG envelopes after time normalization of each repetition on subject-specific mean duration. TCI was then calculated as:
  $$ TC{I}_i=\frac{PS{D_{EMG}}_i\mid {f}_x+ PS{D_{EMG}}_i\mid {f}_y}{PS{D_{EMG}}_i} $$

Where *i* indicates the considered muscle, *PSD* is the power spectral density, *f*_*x*_ and *f*_*y*_ are the frequencies corresponding to the peak of the spectrum of the X and Y coordinates of the fingertip [26]. TCI ranges from 0 (no match between kinematic components and harmonic components within muscle activity) to 1 (complete match between kinematic components and harmonic components within muscle activity). Specifically, we focused on the task principal muscles. The most task-related EMG activations in the present dataset among patients and healthy subjects were AD, PD and BIC profiles (see Results below). This result confirmed previous findings on the same task, showing AD, PD and BIC as the task principal muscles with a TCI > 0.5 in healthy subjects [26]. With learning, TCI values should increase, towards more functional task-related muscular patterns.

### Statistics

A linear mixed model analysis on each outcome measure was applied with dystonia (primary or secondary), block (BF+ or BF-), day (D1 or D5) as fixed effects, “day by block” and “dystonia by day by block” as interaction effects, and subject as random effect. The BAD score of each subject was used in the model as covariate. In particular, the analysis was performed on the following outcome measures: i) Time*error; ii) *CV*_*speed*_; iii) *Dissimilarity* index for proximal joints (elbow and shoulder); iv) *Repeatability*; v) *TCI* for each of the three principal muscles.

Afterwards, the linear mixed model analysis was repeated on the same outcomes but considering the primary and secondary dystonia subjects, separately. In this case, the model used day and block as fixed effects, “day by block” as interaction effect, and BAD score as covariate.

The effect size of each outcome measure was also calculated for each block (BF+ and BF-) and group (primary and secondary dystonia) as the ratio between pre and post change (in the direction of improvement) and the pooled standard deviation of values at D1 and D5.

The statistical analysis was performed in SPSS (IBM) v24.

## Results

Table 1 reports the clinical and demographic details of the recruited patients, as well as the training parameters (tested arm, size of the Figure-8, target speeds and target muscle).

**Table 1:** Clinical and demographic details as well as training parameters of the patients recruited for the study

Subject	Age [years]	Sex	Dystonia	Drugs / DBS^a^	Tested arm	BAD arm^b^	Figure-8 size (circle radius [cm])	Target Speeds [bpm]^c^	BF target muscle
P1	10	F	I	Trihexyphenidyl	Right	1	4	100; 80; 60	AD
P2	10	F	I	Trihexyphenidyl	Right	1	4	80; 60; 50	FCU
P3	16	M	I	None	Right	1	4	100; 80; 60	AD
P4	17	F	I	DBS	Right	1	4	100; 80; 60	FCU
P5	19	M	I	Trihexyphenidyl	Right	1	4	100; 80; 60	ECR
P6	8	M	I	Trihexyphenidyl	Right	1	4	80; 60; 50	FCU
P7	8	M	I	L-Dopa/Carbidopa	Right	1	4	100; 80; 60	FCU
S1	14	M	II	None	Right	2	4	60; 50; 40	LD
S2	10	M	II	None	Right	2	4	60; 50; 40	ECR
S3	8	M	II	None	Right	1	4	20	ECR
S4	16	F	II	DBS	Right	3	7	uncontrolled	BIC
S5	13	M	II	Trihexyphenidyl	Left	3	4	40; 30; 20	LD
S6	6	F	II	None	Right	1	4	80; 60; 40	FCU
S7	8	F	II	Trihexyphenidyl	Right	1	7	30; 40	AD

a) DBS: Deep Brain Stimulation. b) BAD of the dominant/tested arm: 0 (absent) - 4 (severe). c) Among target speed, the speed used for training is highlighted in bold.

From the BAD values it can be noticed that overall children and adolescents with primary dystonia were less impaired than peers with secondary dystonia: all primary dystonic subjects had 1 as BAD score for the tested arm, while secondary ranged from 1 to 3. This difference in severity reflected in the task parameters: all the secondary dystonia subjects were asked to keep lower speeds than primary; some were even not able to keep 3 different speed levels and performed the task at an uncontrolled speed (S4) or at a lower single speed (S3). Finally, for one of the two most impaired subjects (S4) as well as for S7 a larger size of figure-8 was used to make the task feasible (radius of the circle equal to 7 cm). From the randomization order of the blocks, it came out that 4 out of 7 patients with primary dystonia performed BF+ first, then BF-; while among patients with secondary dystonia 3 out of 7 patients used BF in the first week.

The healthy control group consisted of 9 subjects (5 males and 4 females) with a mean age of 15.7 ± 2.8 years. For them, the highest speed values (100; 80; 60 bpm) and the smaller size of the figure-8 were used.

**Fig. 3:**
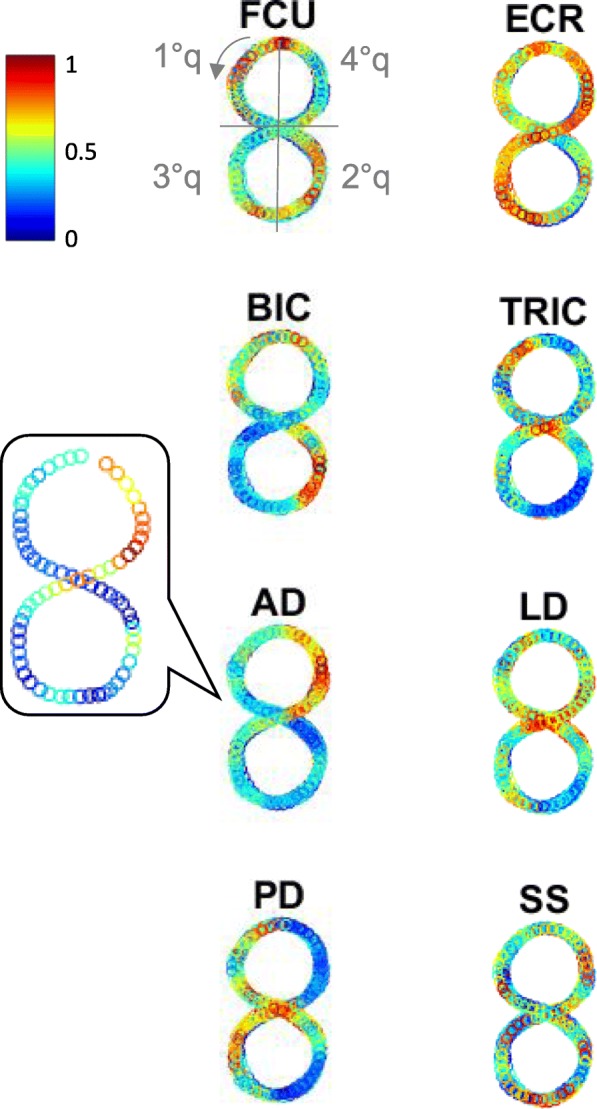
EMG signals along figure-8 trace. Example of EMG activations of one healthy subject for one target speed (30 bpm). They are mapped on the output task (figure-8 trace). In all the overlapped repetitions, each EMG profile envelope is normalized on the maximum in that repetition. Each empty circle is the mean value of the EMG normalized profile in small time windows whose width is calibrated to create a downsampling to match the kinematic sampling frequency (102 samples, to downsample from 2048 Hz to 20 Hz). One single repetition is shown in the inset for AD. The colormap ranges from blue (low muscle activation) to red (high muscle activation). The direction of the drawing and the consequent four quarters of the figure-8 are depicted

The data analysis aimed at investigating kinematics and muscular activations (Fig. 1c and d), as well as their coupling. In all EMG envelopes, the different figure-8 repetitions could be identified, with one or more peaks of different amplitudes for each repetition (Fig. 1d). Figure 3 reports a direct mapping of normalized EMG envelopes on the figure-8 shape, for one representative healthy subject. The EMG envelope of each muscle was time-aligned with the 2D finger trajectory, overlapping all the repetitions carried out at one speed. The colormap allows visualization of the contribution of muscle activity to the specific phases of the figure-8, where red corresponds to the relative maximum activity of that muscle. The most correlated muscles were robustly associated to specific figure-8 phases: BIC showed one main peak for each repetition, in the second quarter of the figure-8; AD exhibited one very clear peak for each repetition in the last quarter; finally, the PD presented two peaks, in the first and third quarters. These three muscles were the main drivers to complete the four quarters of the figure-8: basically, the first quarter was done by PD with a contribution of BIC, the second one by BIC, the third one by PD and the last quarter by AD. Concerning the other muscles, TRIC showed consistent patterns antagonist to BIC: its minimum matched with BIC maximum. LD co-activated both with AD and PD; indeed, LD minimum occurred in the second quarter during which AD as well as PD were not recruited. SS was not strongly modulated along the figure-8 phases. Finally, the most distal muscles FCU and ECR were antagonist, even if without clear and repeatable activation and deactivation peaks for each figure-8 repetition.

**Fig. 4:**
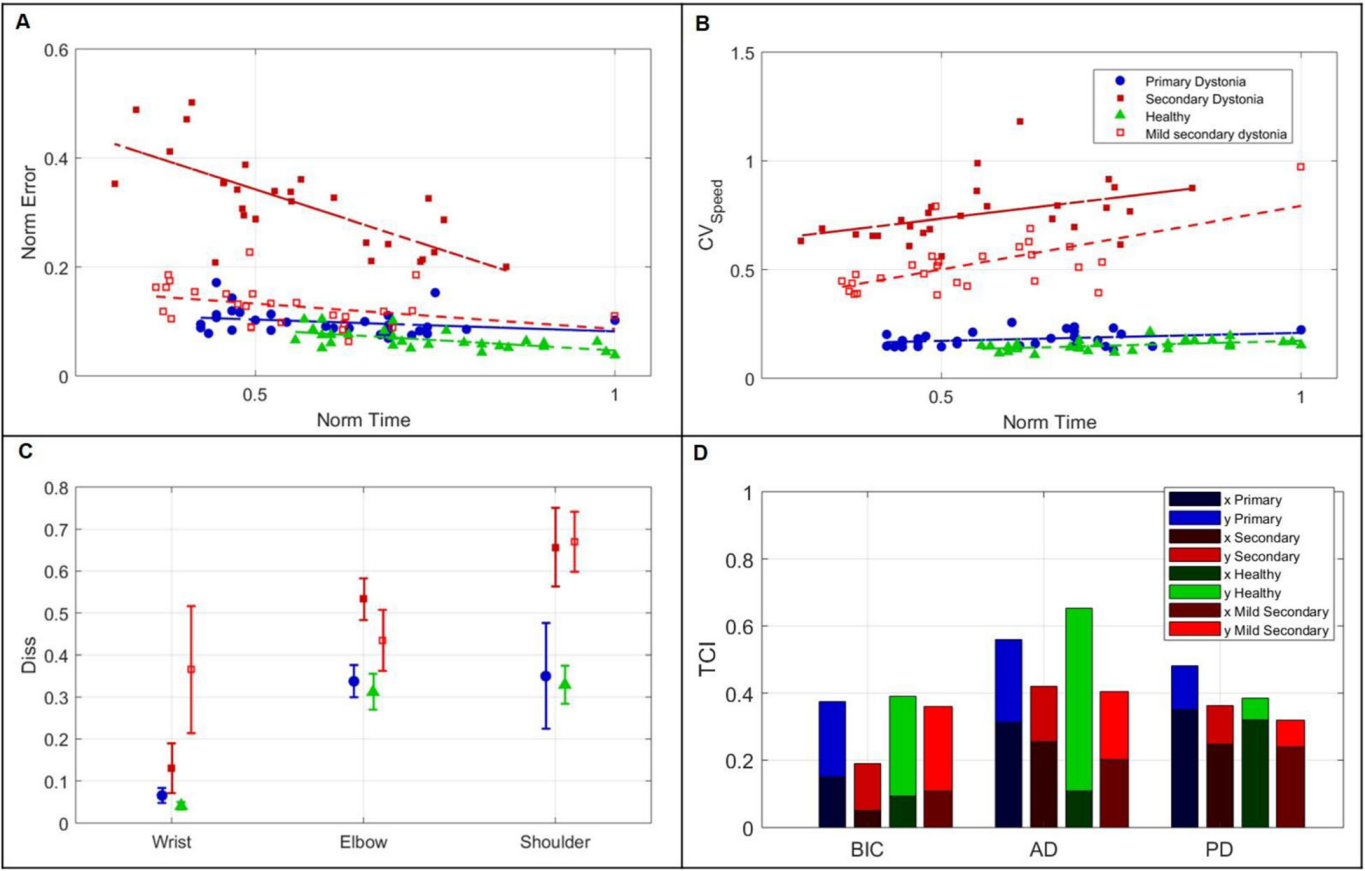
Example of outcome measures of the kinematic and muscular performance collected in four representative subjects. For each outcome measure, one example for one healthy subject, one primary dystonia (P4), one severe secondary dystonia (S5) and one mild secondary dystonia (S1) are reported, in green, in blue, in dark red and in light red, respectively. **a**) SATO, as normalized Error versus normalized Time. Each of the 30 points represents one repetition of figure-8. The linear regression is depicted as dashed lines. **b**) *CV*_*speed*_ as function of the normalized time. Each of the 30 points represents one figure-8 repetition. The linear regression is depicted as dashed lines. **c**) Dissimilarity (0–1) of wrist, elbow, and shoulder trajectories (mean and standard among the 30 repetitions of each subject). **d**) *TCI* indices for the three principal muscles (BIC, AD, and PD). Each muscle is reported as a stacked bar of x and y components (mean values among the three series (3 × 10 repetitions) for each subject)

The computed indices synthetize the kinematic and muscle behavior, taking into account multiple aspects. Figure 4 reports the outcome variables for four representative subjects: one healthy, one with primary dystonia, and two with secondary dystonia (one more severe, one milder). As expected from physiological SATO, spatial accuracy error decreased with a decreased movement execution time (Fig. 4a). The reported subject for severe secondary dystonia group (in dark red) showed this trend; the mild secondary dystonia subject (light red), the primary dystonia subject (in blue) and the healthy one (in green) showed a lower modulation of the accuracy error as a function of movement execution, indicating that the accuracy error saturated to close to the minimal possible value already at the highest speed. A trend towards this accuracy saturation was consistent with the severity level of the four subjects, from severe secondary dystonia to healthy. Figure 4b reports the *CV*_*speed*_ as function of the movement execution. The coefficient of variation should increase with an increased movement execution time. This trend was more visible in the subject with severe secondary dystonia (in dark red), who was more compromised; an intermediate trend was detectable in the mild secondary dystonia (in light red), while a flat trend regardless of the execution time was found for the healthy subject (in green) and the primary dystonia subject (in blue). Figure 4c shows the indices about *dissimilarity*, with a progressive loss of the task shape from distal (wrist) to proximal joints (shoulder). The reported subjects for secondary dystonia were strongly compromised, as indicated by the higher dissimilarity values. Furthermore, the intra-subject variability, represented by the bar indicating the standard deviation among repetitions, was higher for the subjects with dystonia than for the healthy control. Finally, Fig. 4d shows the *TCI* index for the task principal muscles. The healthy subject had the highest functional correlation for the AD activation pattern, with the main contribution along the y-axis (i.e. one peak for each figure-8, as shown in Fig. 3); BIC pattern mainly contributed in the y-direction as well, whereas PD along the x-axis (i.e. two peaks for each figure-8, as reported in Fig. 3). The subject with primary dystonia had a behavior comparable to the one of the healthy control, while the subjects with secondary dystonia had muscular patterns less correlated with the kinematic output and with less clear association to the frequency components (x or y- axes). The milder secondary dystonia subject showed less functional muscular patterns at proximal level (AD and PD), while the BIC activated in a “healthy” way.

All subjects’ outcome measures are reported in Table 2. Overall, patients showed values worse than the corresponding healthy reference values. These quantitative alterations were consistent among outcome measures, i.e. more compromised muscular patterns yielded to a more pronounced deficit in inter-joint coordination and hence a less effective outcome in terms of figure-8 smoothness and repeatability, and of trade-off among accuracy and execution time. Moreover, these outcomes confirmed the aforementioned clinical observations about the motor impairment of the two groups of subjects (Table 1): the values were farther from control values in secondary dystonia than in primary, i.e. children with secondary dystonia were characterized by a more impaired movement performance. This difference was found despite the lower level of difficulty set for the secondary dystonic patients (see Table 1).

**Table 2:** Results of the statistical analysis

	Healthy subjects	Patients Group	BF+		BF-		*P*-value (Day)^a^	*P*-value (Day x Block)^a^	*P*-value (Dystonia)^b^	*P*-value (Day) ^b^	*P*-value (Day x Block)^b^	P-value (Dystonia x Day x Block)^b^
			D1	D5	D1	D5						
Time*Error ↓	0.052 (0.009)	I	0.068 (0.014)	0.057 (0.009)	0.061 (0.01)	0.056 (0.004)	< 0.001	0.050	0.965	0.002	0.700	0.136
		II	0.109 (0.067)	0.107 (0.056)	0.117 (0.071)	0.114 (0.079)	0.821	0.567				
CV_speed_ ↓	0.162 (0.023)	I	0.258 (0.174)	0.246 (0.1)	0.244 (0.073)	0.205 (0.061)	< 0.001	0.001	0.019	< 0.001	0.061	0.258
		II	0.675 (0.267)	0.614 (0.285)	0.74 (0.296)	0.663 (0.227)	< 0.001	0.708				
Diss_EL_ ↓	0.358 (0.075)	I	0.376 (0.104)	0.372 (0.113)	0.362 (0.095)	0.362 (0.098)	0.942	0.129	0.764	0.007	0.004	0.257
		II	0.43 (0.092)	0.391 (0.107)	0.441 (0.186)	0.422 (0.088)	0.002	0.022				
Diss_SHO_ ↓	0.345 (0.068)	I	0.424 (0.129)	0.374 (0.113)	0.397 (0.104)	0.363 (0.037)	< 0.001	0.125	0.154	< 0.001	0.004	0.209
		II	0.552 (0.134)	0.497 (0.198)	0.574 (0.215)	0.553 (0.152)	0.040	0.023				
Repeatability (%) ↑	97.5 (1.2)	I	93.3 (5.4)	95.2 (1.6)	95 (1.9)	94.8 (3.6)	0.080	0.521	0.005	0.050	0.710	0.310
		II	84.8 (6.6)	84.4 (9.7)	79.7 (5.8)	81 (8)	0.253	0.465				
TCI_BIC_ ↑	0.423 (0.109)	I	0.40 (0.107)	0.414 (0.209)	0.444 (0.225)	0.452 (0.113)	0.502	0.877	0.098	0.414	0.175	0.124
		II	0.254 (0.137)	0.269 (0.082)	0.343 (0.118)	0.199 (0.118)	0.135	0.078				
TCI_AD_ ↑	0.66 (0.102)	I	0.522 (0.104)	0.558 (0.114)	0.564 (0.099)	0.583 (0.132)	0.061	0.857	0.599	0.004	0.028	0.046
		II	0.399 (0.188)	0.542 (0.11)	0.404 (0.172)	0.325 (0.185)	0.040	0.018				
TCI_PD_ ↑	0.405 (0.15)	I	0.397 (0.109)	0.429 (0.125)	0.422 (0.183)	0.435 (0.094)	0.103	0.928	0.193	0.579	0.291	0.242
		II	0.287 (0.082)	0.336 (0.081)	0.337 (0.084)	0.238 (0.108)	0.558	0.168				

a) Linear mixed-model considering separately primary and secondary dystonia subjects. b) Linear mixed-model considering the whole sample. In red, *p* < 0.05

The statistical analysis taking into account all patients with the BAD score as covariate showed that the secondary dystonia children carried out the task with a significantly higher *CV*_*speed*_ (*p* = 0.019) and a significantly lower finger outcome repeatability (*p* = 0.005) than primary subjects. Then, to highlight the learning effect, the analysis was focused on the effect of the factor “Day” on all outcomes and how much the Day effect was dependent on the Block (using or not the BF device during training). Considering the whole sample, we observed an overall learning effect in terms of kinematics and muscular indices (Time*Error: p(Dystonia) = 0.002; *CV*_*speed*_: p(Dystonia) < 0.001; *Diss*_*EL*_: *p* = 0.007; *Diss*_*SHOU*_: *p* < 0.001; *TCI*_*AD*_: *p* = 0.004). The AD pattern and the joint coordination showed also a BF-modulated learning behavior (“Day by Block” - *Diss*_*EL*_: *p* = 0.004; *Diss*_*SHOU*_: *p* = 0.004; *TCI*_*AD*_: *p* = 0.028). Finally, the *TCI*_*AD*_ was strongly modulated even when investigating the triple interactive effect (“Dystonia by Day by Block”; *TCI*_*AD*_: *p* = 0.046). This result suggested that the task-related activation of the AD underwent a learning mechanism, significantly modulated by the BF, and with different trend depending on the dystonia type.

When the two patient groups were analyzed separately, both groups significantly improved their performance with training (Primary dystonia: p(Day) < 0.001 for Time*Error, *CV*_*speed*_ and *Diss*_*SHO*_; Secondary dystonia: p(Day) < 0.001 for *CV*_*speed*_, p(Day) = 0.002 for *Diss*_*EL*_, p(Day) = 0.04 for *Diss*_*SHO*_, p(Day) = 0.04 for *TCI*_*AD*_). Instead, the two groups showed a different behavior due to the use of the BF device: a BF-driven learning effect emerged only in the secondary dystonia group as suggested by the significant “Day by Block” interaction effect found for *Diss*_*EL*_ (*p* = 0.022), *Diss*_*SHO*_ (*p* = 0.023), and *TCI*_*AD*_ (*p* = 0.018). The only significant “Day by Block” effect found for primary dystonia was detected on *CV*_*speed*_ suggesting possible worsening of learning with BF in this group. All these findings emerged despite the high inter-subject and intra-subject variability, especially for TCIs.

Table 3 reports the results of the effect size analysis. On average, in secondary dystonia, the use of BF induced an improvement with a small to large effect size on 5 outcome measures (*CV*_*speed*_, *Diss*_*EL*_, *Diss*_*SHO*_, *TCI*_*AD*_, *TCI*_*PD*_). The largest effect size was relative to *TCI*_*AD*_, confirming the results of the statistical analysis. Conversely, when the BF was not used, only one outcome measure (*CV*_*speed*_) showed an improvement with a small effect size. In primary dystonia, a large effect size emerged for Time*Error after the use of the BF, but the same outcome showed a medium effect size even when the BF was not used. The other detectable effect sizes were comparable in BF+ and BF- conditions; for *CV*_*speed*_ effect size was relevant only in BF- block.

**Table 3:** Results of the effect size analysis

Patients Group	Block	Time*Error	CV_speed_	Diss_EL_	Diss_SHO_	Repeatability	TCI_BIC_	TCI_AD_	TCI_PD_
Primary	*BF+*	0.915	0.084	0.045	0.407	0.463	0.074	0.326	0.272
	*BF-*	0.680	0.581	−0.144	0.348	0.256	0.199	0.247	0.255
Secondary	*BF+*	0.042	0.221	0.390	0.324	−0.049	0.130	0.929	0.605
	*BF-*	0.040	0.293	−0.308	0.114	0.185	−1.222	−0.192	−0.756

In orange, large effect size (> 0.8); in blue medium (0.5–0.8); in light grey small (0.2–0.5).

**Fig. 5:**
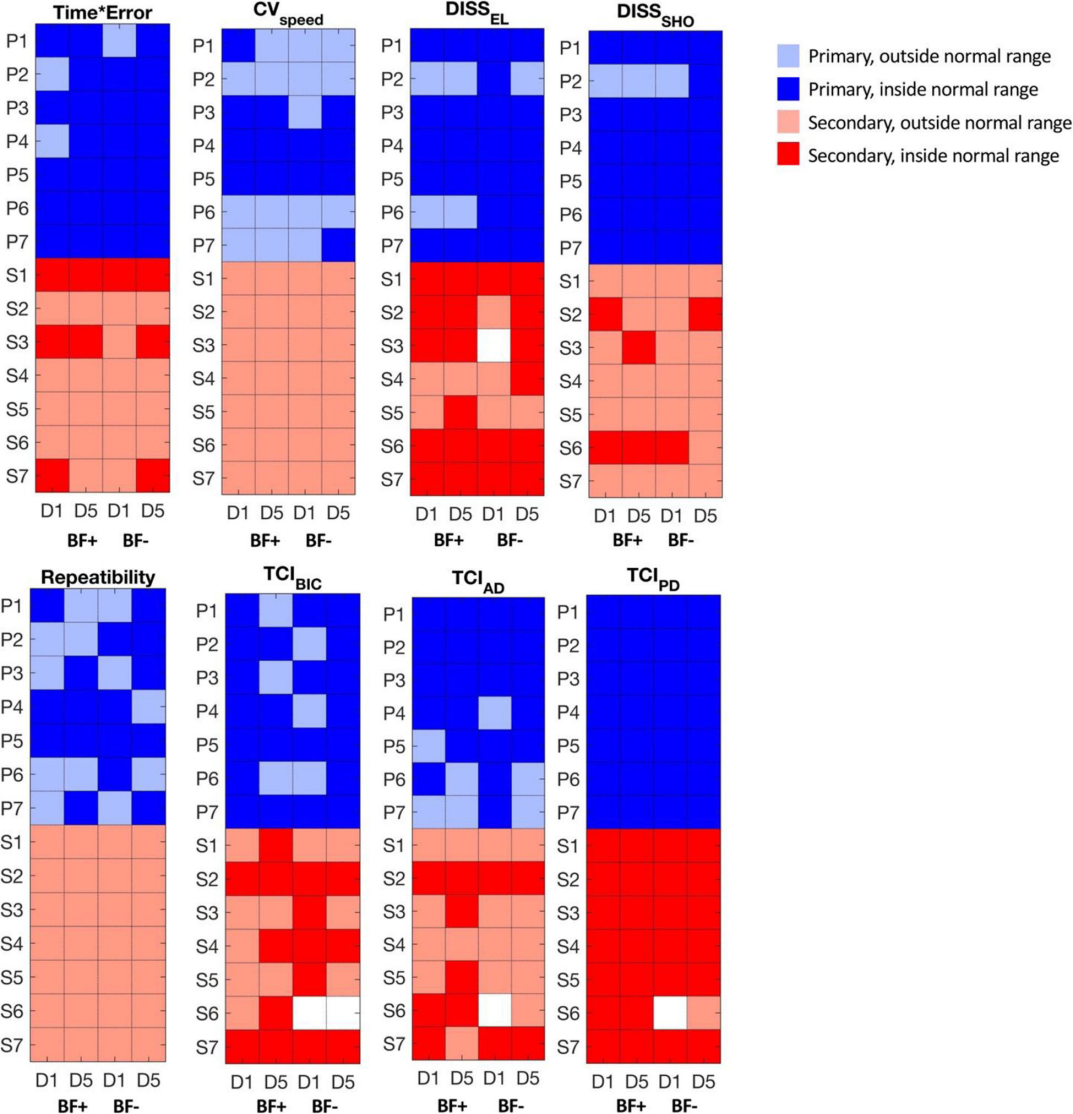
Comparison between dystonia patients and healthy subjects. For each outcome measure, a normality range was identified as the 95% confidential internal achieved by the group of healthy subjects. Mean values obtained by patients in each single session were compared to the normality range and used to represent a map: darker color indicates values within the normality range, lighter color indicates values outside the normality range. White cells indicate data not available due to acquisition failures and/or technical issues

Finally, Fig. 5 reports a colormap to visualize the healthiness of the computed indices for each patient, i.e. if the value is within the range of the healthy control group. Again, it is evident that the severity was greater for the secondary dystonia group. Moreover, in some cases, the learning process between D1 and D5, moved the indices into the healthy range. Specifically, normalization occurred only when BF was used between D1 and D5 for the secondary dystonia group: for *TCI*_*BIC*_ in subjects S1, S4 and S6, for *TCI*_*AD*_ in subjects S3 and S5, for *Diss*_*EL*_ in subject S5, and for *Diss*_*SHO*_ in subject S3. In other cases, indices initially outside the healthy range improved but did not normalize. In a few cases, the indices became worse: *Diss*_*SHO*_ for S2 and *TCI*_*AD*_ for S7.

## Discussion

The current work presents the results of using an EMG-based vibro-tactile biofeedback device during motor training in children and adolescents with primary and secondary dystonia. This study is part of a larger multi-center clinical trial that investigates the efficacy of short- and long-term biofeedback training in this movement disorder. The sensory biofeedback is likely to be integrated into the sensorimotor loop, thus affects both motor performance and learning: learning cannot occur without sensory information to reflect results of performance and to call attention to important elements of the task.

The figure-8 task sheds lights on multiple aspects of the subject-specific movement strategy, separating the kinematic and electromyographic task-related components from task-unrelated components. This task allows a frequency analysis of the coupling between kinematic and EMG signals [26]. The defined outcome measures capture the trade-off between execution time and accuracy, the velocity-dependent smoothness, the movement repeatability, the loss of the figure-8 shape from distal to proximal joints, and the task-correlated muscle activity. Indeed, all muscle patterns were correlated to some degree with the kinematics of the figure-8 shape, depending on the signal noise, on unwanted components, and on the subject-specific muscular strategy.

The findings of this study are consistent with the prediction of our hypothesis that the use of the biofeedback device promotes a more rapid and effective learning with practice in secondary dystonia compared to primary dystonia. In particular, a significant improvement of muscular recruitment (increased task-correlation of the task principal muscles’ activity) with a medium to large effect size was evident in the secondary dystonia group after BF training, suggesting the reduction of unwanted and noisy components. The large to medium effect sizes related to the functional activation of the proximal muscles in secondary dystonia when the BF was used are promising results, considering the small sample size of this pilot study. Such results translated into an improvement of the kinematics of the upper joints, although with a small effect size: especially, the proximal joints of the limb “serial chain” showed more functional motions, i.e. the output desired shape was more represented at all involved degrees of freedom. Such enhancement of the muscular-kinematic task-correlation consistently corresponded to an improvement in the final performance. Indeed, a trend, although not significant, of improvement in the speed-accuracy trade-off was achieved by decreasing the accuracy error.

The mechanism underlying this learning might be due to the increased implicit awareness of the activity of the target muscle (i.e. the most responsible for movement errors) induced by the EMG-based vibro-tactile biofeedback. By focusing attention, the causative muscle might be preferentially trained, leading to improved patterns of movement.

The study results confirm the preliminary findings on few subjects that had reported a positive qualitative effect of the biofeedback training on the writing outcome, while no learning was achieved when children practiced for the same amount of time without wearing the biofeedback device [24].

Although subjects with primary dystonia do not appear to have sensory impairment, the biofeedback approach could have been useful for this group to guide learning or to better refine movements. However, our results showed that the biofeedback-aided training was not useful in accelerating or improving motor learning in subjects with primary dystonia. One could claim that the difference in improvement between primary and secondary dystonia was due to a ceiling effect in primary dystonia, but the statistical analysis highlighted that, despite the better initial motor performance, subjects with primary dystonia achieved a significant learning of the motor task, regardless the additional sensory information. It is therefore likely that the scaled vibration of the biofeedback device represents a redundant or unnecessary signal added to the already properly functioning sensory information in primary dystonia [31] [12]. Therefore, we can support the theory of the failure of motor learning, which states that, even for the simplest tasks, learning fails when the results of a particular movement cannot be well detected by the controller [11]. In this framework, biofeedback techniques can be leveraged for children and adolescents with secondary dystonia to redirect attention to a particular sensory representation, focusing on errors that might have been otherwise ignored [11, 32].

The study has some limitations. First of all, it recruited a limited number of subjects (7 with primary dystonia and 7 with secondary dystonia). A larger sample size is needed to derive final conclusions about the theory of the failure of motor learning in secondary dystonia subjects. Secondly, the choice of different target muscles and customized difficulty levels for each subject might have added variability in the study results. Thirdly, our results could be influenced by the different level of impairment of the two groups, with secondary dystonia subjects being more compromised than primary peers. The subject-specific difficulty level partially compensated for the different degree of impairment; however primary subjects still exhibited a better performance. In future, less impaired subjects should be challenged with higher difficulty levels (higher speed) in order to minimize the ceiling effect which was visible in some primary dystonia subjects. Lastly, a group of healthy subjects should be involved in the complete protocol in order to investigate the effect of the biofeedback device on motor learning in subjects with an intact sensory-motor loop. The hypothesis is that healthy subjects exhibit a similar behavior as primary dystonia subjects, with a learning effect only due to practice, not mediated by the use of the biofeedback device.

The ongoing multi-center clinical trial will overcome some of these limitations: more subjects will be recruited and the learning effect on healthy controls will be investigated. Furthermore, the effect of the device on a second task, a back and forth spoon self-feeding task, will be analyzed. Finally, the long-term effect of the use of the biofeedback device during daily life activities (wearing the device at least 5 h a day for 1 month) will be investigated. While short-term biofeedback may bring about improved awareness of ongoing movement, long-term use has the potential to facilitate plasticity of the neural pathways that encode motor commands. Therefore, scaled vibratory feedback may strengthen the cortical representations associated with the motor tasks.

## Conclusions

Overall, this work sheds lights on the potential effectiveness of sensory biofeedback training in helping children and adolescents with dystonia to gain improved control over specific muscles during voluntary motion. Since subjects with secondary dystonia are known to have sensory deficits [12, 13], and this study has shown that augmentation of sensory function improves motor learning, our results are consistent with the hypothesis from the theory of failure of motor learning, that sensory deficits in secondary dystonia perpetuate motor deficits by impairing motor learning. In this study, the primary dystonia subjects function as an important control group to show that in the absence of sensory deficits, the sensory biofeedback does not have an independent effect on dystonia.

Symptoms of dystonia are highly-disabling and strongly influence function of everyday life, from school activities to social interaction. Therefore, these results may further support the use of biofeedback as an effective noninvasive intervention in children and adolescents with secondary dystonia. The use of a small wearable device, which can be easily disguised in clothes, makes the intervention suitable for long-term use in daily contexts.

## Data Availability

The datasets used and/or analyzed during the current study are available from the corresponding author on reasonable request.

## Abbreviations

AD: Anterior Deltoid
BAD: Barry-Albright Dystonia Scale
BF: Biofeedback
BIC: Biceps Brachii
CP: Cerebral palsy
DBS: Deep Brain Stimulation
ECR: Extensor Carpi Radialis
EMG: ElectroMyoGraphy
FCR: Flexor Carpi Radialis
LD: Lateral Deltoid
PC: Principal Components
PCA: Principal Component Analysis
PD: Posterior Deltoid
PSD: Power spectral density
SATO: Speed-accuracy trade-off.
SS: Supraspinatus
TCI: Task Correlation Index
TRIC: Triceps Brachii
